# Bax inhibitor 1 preserves mitochondrial homeostasis in acute kidney injury through promoting mitochondrial retention of PHB2

**DOI:** 10.7150/thno.40098

**Published:** 2020-01-01

**Authors:** Jin Wang, Pingjun Zhu, Ruibing Li, Jun Ren, Yingmei Zhang, Hao Zhou

**Affiliations:** 1 Chinese PLA General Hospital, Medical School of Chinese PLA, Beijing, 100853, China.; 2 Center for Cardiovascular Research and Alternative Medicine, University of Wyoming College of Health Sciences, Laramie, WY 82071 USA.; 3 Department of Cardiology and Shanghai Institute of Cardiovascular Diseases, Zhongshan Hospital, Fudan University, Shanghai, China 200032.

**Keywords:** BI1, mitochondria, tubule cells, AKI, PHB2.

## Abstract

Bax inhibitor-1 (BI1) conveys anti-apoptotic signals for mitochondria while prohibitin 2 (PHB2) is implicated in sustaining mitochondrial morphology and function. However, their regulatory roles in acute kidney injury (AKI) are largely unknown.

**Methods:** In human patients with AKI, levels of BI1 in urine and plasma were determined using ELISA. An experimental model of AKI was established using ATP depletion-mediated metabolic stress and ischemia-reperfusion injury (IRI) in primary tubule cells and *BI1* transgenic mice, respectively. Western blots, ELISA, qPCR, immunofluorescence, RNA silencing, and domain deletion assay were employed to evaluate the roles of BI1 and PHB2 in the preservation of mitochondrial integrity.

**Results:** Levels of BI1 in urine and plasma were decreased in patients with AKI and its expression correlated inversely with renal function. However, reconstitution of *BI1* in a murine AKI model was capable of alleviating renal failure, inflammation and tubular death. Further molecular scrutiny revealed that BI1 preserved mitochondrial genetic integrity, reduced mitochondrial oxidative stress, promoted mitochondrial respiration, inhibited excessive mitochondrial fission, improved mitophagy and suppressed mitochondrial apoptosis. Intriguingly, levels of the mitochondria-localized PHB2 were sustained by BI1 and knockdown of PHB2 abolished the mitochondrial- and renal- protective properties of BI1. Furthermore, BI1 promoted PHB2 retention within mitochondria through direct interaction with cytoplasmic PHB2 to facilitate its mitochondrial import. This was confirmed by the observation that the C-terminus of BI1 and the PHB domain of PHB2 were required for the BI1-PHB2 cross-linking.

**Conclusion:** Our data have unveiled an essential role of BI1 as a master regulator of renal tubule function through sustaining mitochondrial localization of PHB2, revealing novel therapeutic promises against AKI.

## Introduction

Tubule cell death and later proinflammatory response are well acknowledged as the predominant histological features of various forms of acute kidney injury (AKI) - a critical factor in the etiology of chronic kidney disease 1, 2. Although the intrinsic cell death events may initiate at distinct cellular locations, many of them ultimately converge on mitochondria to turn on the common intrinsic mitochondrial apoptosis cascade 3. A preliminary screening of potential regulators governing mitochondrial protection led to the identification of a protein with apparently anti-mitochondrial apoptosis activity which hence was termed Bax inhibitor-1 (BI1). Although BI1 is an endoplasmic reticulum (ER)-localized cell death suppressor 4, it actively participates in multiple pathophysiological processes in mitochondria 5. At the early stage of apoptosis, BI1 blocks Bax-related mitochondrial outer membrane (MOM) permeabilization to retard initiation of mitochondrial apoptosis 6. Moreover, BI1 may also directly interact with Bcl2 and/or Bcl-XL to augment mitochondrial anti-apoptotic signals 7. In addition to apoptosis, other cellular events such as mitochondrial calcium intake 8, mitochondrial ROS production 9, and bioenergetics^10^ are also under the regulation of BI1. The indispensable role of BI1 in ensuring mitochondrial homeostasis was validated by our recent work where BI1 overexpression renders resistance of myocardial microvasculature to ischemia-reperfusion injury through inhibiting mitochondrial fission and preventing cardiomyocyte apoptosis 5, 11. This evidence indicates the permissive role of BI1 in the maintenance of mitochondrial quality whereas the mechanistic connection and functional relationship between BI1 and mitochondrial homeostasis in AKI remain elusive.

Recent advance in the time-of-flight mass spectrometry and the two-dimensional electrophoretic detection have made it possible for elucidation of proteins targeting or interacting with BI1 12, 13. Among these, prohibitin 2 (PHB2) 13 has been reported to regulate diverse mitochondrial function including mitochondrial respiration modification^14^, mitocondrial criaste perservation^15^ and mitophagy activation^16^. Although PHB2 is found in the nucleus, mitochondria and cytosol, the majority of cellular responses exerted by PHB2 may be attributed to its regulatory function in the mitochondria 17. This notion received support from the observations that PHB2 export from mitochondria into cytoplasm/nucleus is a cardinal triggering mechanism for mitochondrial apoptosis 18, 19. Functional mutants of PHB2 in its mitochondria-localized residues rather than nucleus-localization sequences disturbs cancer proliferation and obligates cells to apoptosis 20, 21. Mechanistically, mitochondria-localized PHB2 maintains mitochondrial respiration chain activity 22, promotes cristae morphogenesis 23, restricts malignant mitochondrial fission^20^. Loss of PHB2 in renal podocytes prompts progressive proteinuria and kidney failure due to the hyperactive insulin/IGF-1 signaling cascade 24. This scenario was also supported by a more recent study that PHB2-mediated mitophagy reduces renal tubule injury through repressing NLRP3 inflammasome activation 25. Despite ample experimental and clinical evidence favoring the functional importance of PHB2 in maintaining renal function, little is known with regards to the structural alteration of mitochondria-localized PHB2. Will PHB2 dissociate from mitochondria in pathological settings, and if so, will that trigger tubular mitochondrial apoptosis in AKI? To this end, the present study was designed to determine whether BI1 could stabilize PHB2 and promote its retention in mitochondria, thus rendering a pro-survival phenotype of mitochondria in renal tubule cells.

## Materials and methods

### Surgical procedures

All experimental procedures described here were approved by the Animal Care and Use Committees of the Chinese PLA General Hospital (Beijing, China) and the University of Wyoming (Laramie, WY, USA). *BI1* transgenic (*BI1^TG^*) mice with C57BL/6 background were generated as described previously 5, 11. Age and sex-matched C57BL/6 mice were used as the WT group. Ischemia AKI was induced using a renal IRI model. In brief, renal pedicles were clamped for 30 minutes and reperfusion was induced for 24h. Sham group received similar operation without renal pedicle clamping 26, 27. To knockdown PHB2 *in vivo*, WT and *BI1^TG^* mice were subjected to daily intravenous injection of scramble control or PHB2 siRNA (Ctrl-si or PHB2-si) three days prior to IRI, as described 28. Renal histology was examined via HE staining. Tubular injury index was determined as reported 29. BUN ELISA kit (MBS751125) and serum creatinine ELISA Kit (MBS2540563) were purchased from MyBioSource, Inc. to detect levels of BUN and Cr after IRI.

### Cell treatment

Primary tubular epithelial cells were isolated from *BI1^TG^* and WT mice. In addition, human proximal tubular epithelial cell line (HK2, ATCC® CRL-2190™) was maintained as described previously 29. ATP depletion-mediated metabolic stress was used to establish the model of *in vitro* mimicked IRI (mIRI) through incubating the primary tubule cells and/or HK2 cells with 10 mM rotenone in glucose-free DMEM for 3-h followed by a 3-h full culture medium incubation.

### Patients and samples collection

Adult patients (n=28) with AKI (defined as ≥1.5-fold increase in serum creatinine in compliance with the RIFLE-Acute Kidney Injury Network criteria) admitted to the intensive care units (ICU) in PLA general hospital were recruited. Control samples were collected from 27 ICU patients who did not develop AKI. The anthropometric information of patients is listed in Table S1. All experimental protocol in this study involving human participants was approved by the Ethics Committee of PLA general Hospital, Beijing, China. All patients or their family expressed their willingness to participate in through an informed consent form. Urine, urinary sediments and blood were collected from the participating subjects. The concentrations of BI1 in urine and plasma were determined using a commercial ELISA kit (Catalogue No. MBS7234646; MyBioSource).

### Electron Microscopy

Cells were fixed with 2.5% glutaraldehyde in 0.1 M cacodylate buffer for 2 h, before being rinsed three times with 0.1 M cacodylate buffer and fixed in 1% osmium tetraoxide for 1 h. Samples were dehydrated by a graded series of ethanol and embedded in araldite. Ultrathin sections were stained with uranyl acetate and lead citrate and examined with an electron microscope (JEM-1200EX, JEOL Co., Japan).

### Immunofluorescence

Kidney frozen tissue sections or cells fixed with 4% formaldehyde were permeabilized with 0.2% Triton X-100 for 5 min. After rinsing with PBS three times, samples were blocked with 10% goat serum for 1 h and were then incubated with primary antibodies (AQP1, a proximal tubular marker, 1:100, Abcam, #ab15080; Tom20, a mitochondria marker, 1:500, Abcam, #ab186734) overnight at 4°C. After washing, secondary antibodies including Alexa 555-conjugated donkey anti-mouse IgG (A31570), Alexa 555-conjugated donkey anti-rabbit IgG (A31572), Alexa 488-conjugated donkey anti-rabbit IgG (A21206) or Alexa 488-conjugated donkey anti-mouse IgG (A21202) from Invitrogen were used. The 4',6-diamidino-2-phenylindole (DAPI) was used for staining of cell nuclei. Florescence was visualized under a confocal microscope using the Nikon NIS-Elements software (Nikon, Tokyo, Japan). Mitochondrial fission was evaluated by cell counts of fragmented mitochondria. To determine immunofluorescence, the immunosignals were converted into average grayscale intensity which was subsequently analyzed using an Image-Pro Plus 6.0 software^30^.

### PHB2 and BI1 transfection

PHB2 possesses 299 amino acids (aa), including a N-terminal mitochondrial targeting domain (N, 1-50 aa), a PHB domain (PHB, 68-185 aa), a coiled coil domain (CC, 190-264 aa), and a C-terminal region (C, 265-299 aa). BI1 contains 237 aa with a N-terminal domain (N, 1-29 aa), several transmembrane domains (TM, 30-222 aa) and a C-terminal domain (C, 223-237 aa). PHB2 and BI1 sequences were amplified from the cDNA of human HK2 cells. Amino acids 51-299 (PHB2ΔN), 1-264 (PHB2ΔC), 1-189 and 265-299aa (PHB2ΔCC), 1-67 and 186-299aa (PHB2ΔPHB) and full length (1-299 aa) of PHB2 were amplified using PCR and the produces were introduced into pcDNA3.1/Myc (Invitrogen) to construct the Myc-PHB2 mutants. Similarly, BI1ΔN (30-237 aa), BI1ΔC (1-222 aa), BI1ΔTM (1-29 and 223-237 aa) and full length (1-237 aa) of BI1 were inserted into pcDNA3.1/HA (Invitrogen) to generate BI1 mutants. siRNAs against BI1 (BI1-si), TIM23 (TIM23-si), and PHB2 (PHB2-si) were designed and synthesized by GenePharma Co, Ltd. (Shanghai, China). The control siRNA (Ctrl-si) was employed as a negative control under similar conditions. Transfection with plasmids or siRNA was performed using Lipofectamine 2000 (Invitrogen) based on our previous studies 31, 32.

### Western blot analysis and co-immunoprecipitation and

Urine specimen was collected and was then centrifuged at 3000 g for 30 min at 4°C. After removal of the supernatant, the urine sediment was used for western blot, as previously described 33, 34. Then, proteins in urine sediment, tissue and cells were electrophoresed by SDS-PAGE and transferred to PVDF membranes (Millipore, ISEQ00010) which were blocked with 5% skim milk. Then, membranes were incubated with primary antibodies at 4°C overnight. The bands were visualized by a Western-Light chemiluminescent detection system (Image Station 4000 MM Pro, XLS180, Kodak, USA) 35. The antibody information was provided in Table S2. Protein interaction was estimated by a Co-Immunoprecipitation Kit (Pierce, 26149) according to our previous procedure 30, 36. The antibody specificity of BI1 was verified through analyzing the expression of BI1 in HK2 cells transfected with si-BI1 and/or scramble RNA (Ctrl-si) (Figure S1A-B).

### Fractionation of mitochondria

Cells were harvested, rinsed with PBS, and suspended in isolation buffer (3 mM Hepes-KOH (pH 7.4), 0.21 M mannitol, 0.07 M sucrose, 0.2 mM EGTA) on ice. Then, homogenates were overlaid on 0.34 M sucrose followed by centrifugation at 500 × g. These steps were repeated for three times before centrifugation of supernatants at 10,000 × g to extract mitochondrial fraction 37. After repeating the centrifugation step for four times, supernatant containing cytosolic fraction was collected. To isolate mitochondrial outer-membrane and mitoplast (inner-membrane plus matrix), isolated mitochondria fraction was resuspended in 0.15 mg/mL digitonin and was then centrifuged at 10,000 × g. Supernatants were collected as outer-membrane fractions, and pellets were collected as mitoplast 38.

### Mitochondrial membrane potential and ROS staining

Mitochondrial potential was analyzed through JC-1 probe (Invitrogen^TM^, T3168) according to manufacturer's protocol. MitoSOX red mitochondrial superoxide indicator (M36008) and CellROX™ Green Reagent (C10444), purchased from Invitrogen, Inc., were used to stain mitochondrial ROS (mito-ROS) and cytoplasmic ROS (cyto-ROS), respectively. For determination of immunofluorescence pictures, the immunosignals were converted into average grayscale intensity which was subsequently analyzed using Image-Pro Plus 6.0 software 39.

### Mitochondrial respiration and mitophagy detection

Mitochondrial respiration was measured through analysis of mitochondrial oxygen consumption rate (OCR), as described previously by our group 36. In brief, cells were seeded at 40 000 cells/well on 96- well XFe96 cell culture microplates and cultured for 48 hours under an XFe96 extracellular flux analyzer (Agilent Technologies). For respiration assays, cells were incubated in a CO_2_- free environment for 1 hour, and OCR was measured every 3 minutes for the next 90 minutes. First, OCR was quantified in basal conditions (20 mmol/L glucose), then with 1 μmol/L oligomycin (ATP synthase inhibitor), next with 0.125 μmol/L FCCP (mitochondrial respiration uncoupler), and finally with 1 μmol/L rotenone/antimycin A (complex I and III inhibitors, respectively). Complex I Activity Assay Kit (ab109721) and Complex II Activity Assay Kit (ab109908), purchased from Abcam, were used to determine the activity of ETCs activity per manufacturer's protocol 40.

Mt-Keima (pMT-mKeima-Red, #AM-V-251, MBL Medical & Biological Laboratories Co., ltd. Woburn, MA) is a ratiometric pH-sensitive fluorescent protein that is targeted into the mitochondrial matrix. Ratio (543/458nm) of mKeima emission light was calculated as an index of mitophagy.

### qPCR

RNA was isolated using RNeasy Mini Kit (Qiagen #74104). Up to 5 μg of total RNA were reverse-transcribed to obtain cDNA using SuperScript III (Invitrogen #18080-051). Quantitative PCR was performed using SYBR Green supermix (Bio-Rad #1725120) per manufacturer's maneul^41^. mtDNA copy was determined through qPCR analysis of the ratio between complex IV and GAPDH segments. NADH dehydrogenase subunit 1 (ND1) and cytochrome c oxidase subunit I (COX-1) transcriptions were used to evaluate mtDNA transcripts. The qPCR primers have been shown in the Table S3.

### Cellular viability detection

Apoptosis in tissue and cell was examined using TUNEL staining (Thermo Fisher Scientific, Inc.) according to the manufacturer's instruction 42. Besides, MTT assay and LDH release assay were determined via commercial kits (No. ab211091 and ab102526) purchased from Abcam, Inc.

### Statistical analysis

Data are presented as Mean ± SEM and were analyzed using a one-way ANOVA using a GraphPad Prism 5.0 software. Associations between groups were determined using the Spearman rank correlation. *P*< 0.05 was considered being statistically significant.

## Results

### BI1 is downregulated in AKI and reconstitution of BI1 attenuates IRI-mediated renal injury

**Figure 1:**
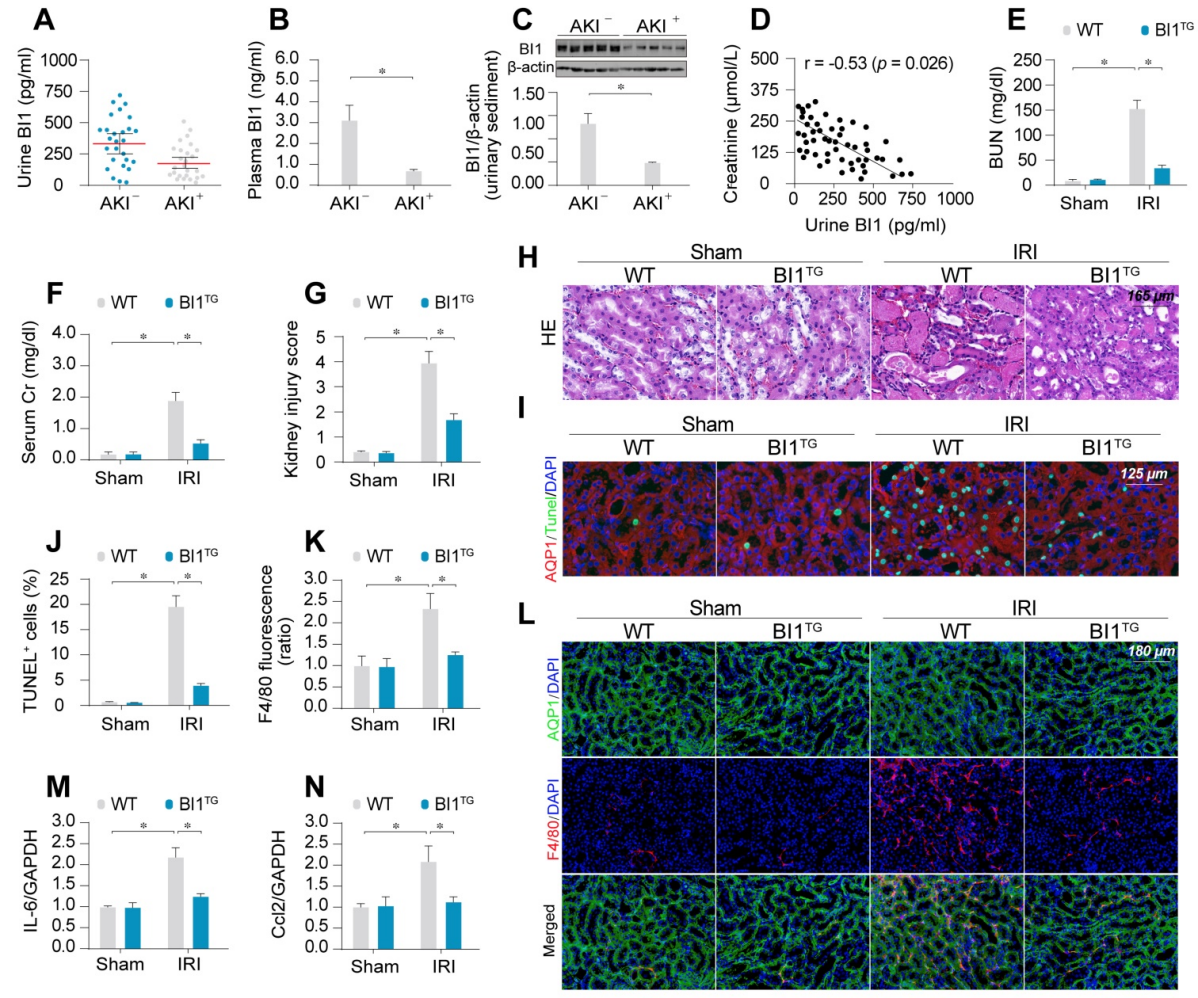
BI1 level is suppressed in ischemic AKI. (**A-B)** BI1 protein levels in urine and plasma from AKI patients using ELISA. **(C)** Urine sediments were collected from patients with AKI or otherwise healthy controls and then western blotting was used to evaluate the expression of BI1 in urine. **(D)** Correlation between urine BI1 content and peak serum creatinine levels in patients with AKI. **(E-F)** Levels of BUN and serum creatinine from BI1 transgenic (*BI1*^TG^) and wild-type (WT) mice subjected to renal ischemia-reperfusion injury (IRI) *in vivo*.** (G-H)** Structural alteration of tubule after IRI using H&E staining. Semi-quantitative analysis of tubular injury (tubular atrophy or dilatation, loss of brush border, vacuolization, epithelial cell shedding, and denuded tubular basement membrane) was scored as: 0, normal; 1, <10%; 2, 10%-25%; 3, 25%-50%; 4, 50%-75%; 5, 75%-100% of affected area from 20 random fields. **(I-J)** Tubule death determined using TUNEL staining. AQP1 was employed to stain proximal tubule. **(K-L)** Immunofluorescence assay for F4/80 pro-inflammatory cells. **(M-N)** RNA was isolated from reperfused kidneys and transcriptions of Ccl2 and IL-6 were determined using qPCR. Experiments were repeated at least three times and data are shown as mean ± SEM (n = 6 mice or 3 independent cell isolations per group). *p<0.05.

To evaluate the potential clinical relevance of BI1 in AKI, urine, urinary sediments and plasma of AKI patients admitted to ICU were analyzed. Compared with ICU patients without AKI, levels of BI1 were found much lower in the urine, urinary sediments and plasma from patients with AKI compared with the non-AKI controls (Figure 1A-C). We next examined the relationship between BI1 levels and renal pathology in a clinical setting. Association analysis revealed an inverse relationship between BI1 and creatinine levels (Figure 1D), suggesting a possible causal relationship for the lower BI1 levels in acute kidney dysfunction.

To better discern the causal relationship between low BI1 levels and AKI pathogenesis, BI1 transgenic (*BI1^TG^*) and their WT littermate mice were subjected to IRI or sham procedure. Baseline reconstitution of *BI1* had no effect on renal function. However, levels of BUN and Cr (Figure 1E-F) were overtly elevated in response to IRI in WT mice, the effect of which was absent in *BI1^TG^* mice. In agreement with the renal function improvement, IRI-induced tubular histological damage was also drastically recovered in *BI1^TG^* mice (Figure 1G-H).

Extensive tubular death is a triggering mechanism for IRI-related renal dysfunction. As shown in Figure 1I-J, IRI overtly enhanced TUNEL-positive apoptotic tubule cells in WT mouse kidneys, the effects of which were greatly attenuated in *BI1^TG^* mice. As a result of cell death, abnormal kidney inflammation as manifested through immunofluorescence of F4/80 interstitial macrophages was enhanced by IRI although the effect was nullified by *BI1* reconstitution (Figure 1K-L). Consistent with these data, IRI-induced augmentation in proinflammatory transcription was also alleviated in *BI1^TG^* mice (Figure 1M-N). *In vitro* study was performed using ATP depletion-mediated metabolic stress to establish a mimicked IRI model (mIRI) in freshly isolated primary tubule cells. Similar to the finding noted *in vivo*, mIRI-mediated cell death (Figure S1C) and proinflammatory response (Figure S1D-E) were also alleviated by BI1 overexpression. Taken together, *BI1* reconstitution was capable of mitigating IRI-related renal pathological phenotypes.

### IRI-evoked mitochondrial damage is reduced by BI1

**Figure 2:**
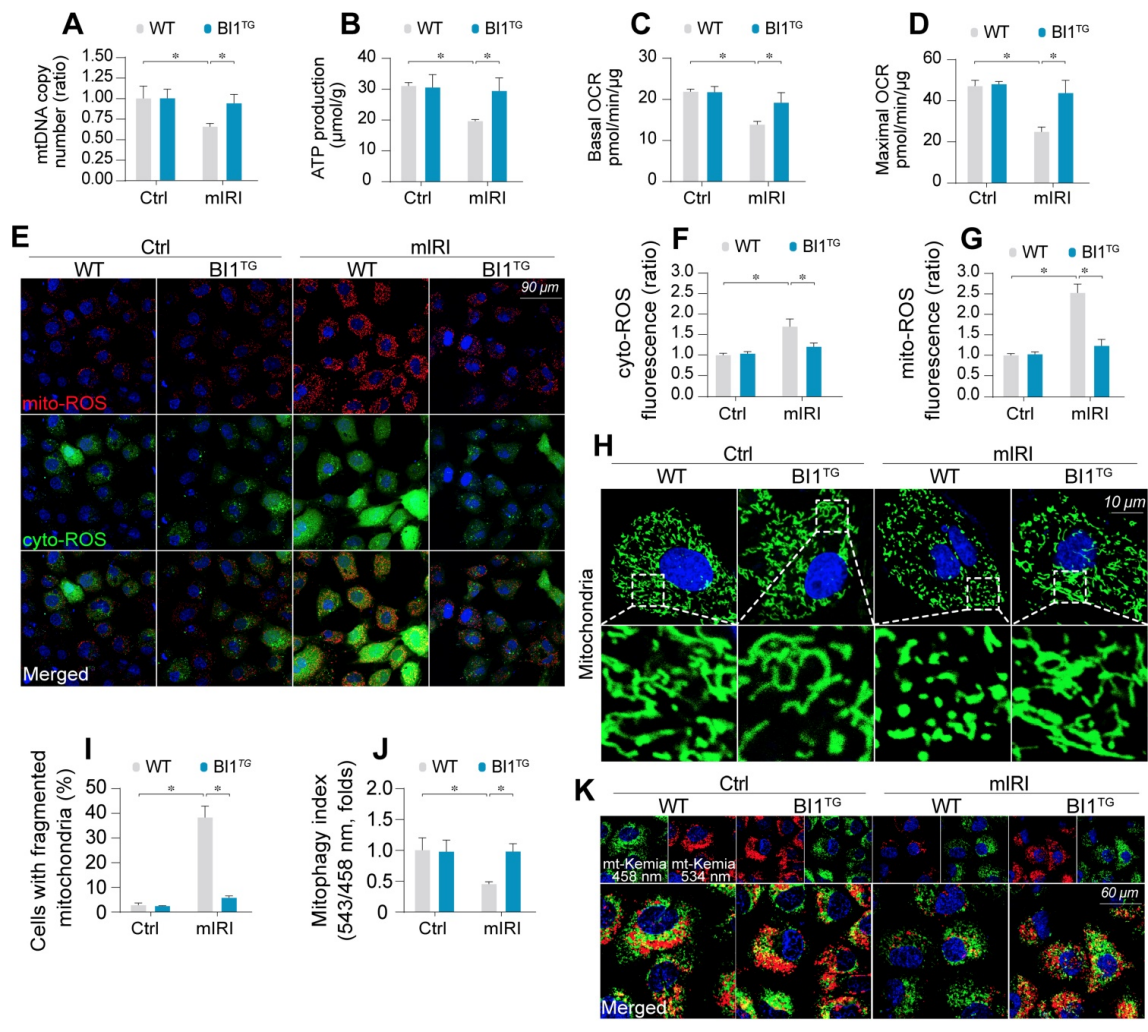
** BI1 overexpression sustains mitochondrial homeostasis under kidney IRI. (A)** mtDNA copy was determined through analysis of the complex IV segment-GAPDH segment ratio using qPCR. **(B)** Cellular ATP production was determined* in vitro*. **(C-D)** Mitochondrial OCR was determined using an XFe96 extracellular flux analyzer. **(E-G)** Levels of mitochondrial ROS (mito-ROS) and cytoplasmic ROS (cyto-ROS) were analyzed using MitoSOX red and CellROX™ green reagent, respectively. **(H-I)** Mitochondrial division was determined using immunofluorescence. Mitochondrial fission was quantified via counting the number of cells with fragmented mitochondria. **(J-K)** mt-Kemia assay for the assessment of acidic mitochondria. The ratio of 534/458 nm was used to quantify the acidic mitochondria index. Experiments were repeated for at least three times and data are shown as mean ± SEM (n = 6 mice or 3 independent cell isolations per group). *p<0.05.

Mitochondrial injury is suggested to serve as a key pathophysiological determinant of AKI 43. Following mIRI challenge, mitochondrial DNA (mtDNA) copy (Figure 2A) and transcription (Figure S2A-B) were repressed in tubule cells isolated from WT mice (WT tubule cells), in parallel with a drop in the activity of mitochondrial electron transport chain complexes (ETCx) primarily encoded by mtDNA (Figure S2C-D). As a result of ETCx dysfunction, mitochondrial respiration, as assessed by ATP production (Figure 2B) and oxygen consumption rate (OCR) (Figure 2C-D), was impaired by mIRI in WT tubule cells. Moreover, the levels of toxic mitochondrial ROS (mito-ROS) and cytoplasmic ROS (cyto-ROS) were upregulated in WT cells under mIRI as expected (Figure 2E-G). Interestingly, *BI1* reconstitution sustained mitochondrial genomic function, improved ETCx activity, recovered mitochondrial respiration and rectified mitochondrial oxidative status.

Mitochondrial function is tightly regulated by morphological integrity. Under normal condition, mitochondria were filamentous whereas mIRI prompted mitochondrial cleavage into several fragmentations (Figure 2H-I). Besides, excessive mitochondrial fission was followed by a decline in mitochondrial biogenesis, as assessed using qPCR analysis for the expression of Sirt3 and PGC1α (Figure S2E-F). In addition, mitophagy evaluated using mt-Keima assay to reflect the amount of acid mitochondria, was also repressed by mIRI due to BI1 downregulation (Figure 2J-K). Interestingly, reconstitution of *BI1* sustained an elongated network of mitochondrial mass, improved mitochondrial biogenesis, and enhanced mitophagy. Furthermore, mitochondrial morphology was evaluated using electron microscopy. As shown in Figure S2G, elongated mitochondrial tubules were found under physiological condition (yellow arrows). However, swollen mitochondria with fractured cristae appeared in response to mIRI stress (red arrows) and this structural alteration was reconciled by BI1 overexpression.

Mitochondrial dysfunction triggers mitochondria-dependent apoptosis featured by Bax translocation onto mitochondria and caspase-9 activation. Data shown in Figure S2H noted that mIRI provoked Bax migration onto mitochondria and caspase-9 activation, the effects of which was abolished by *BI1* reconstitution.

### BI1 enhances PHB2 import into mitochondria via promotion of cytoplasmic PHB2 docking onto MOM

**Figure 3:**
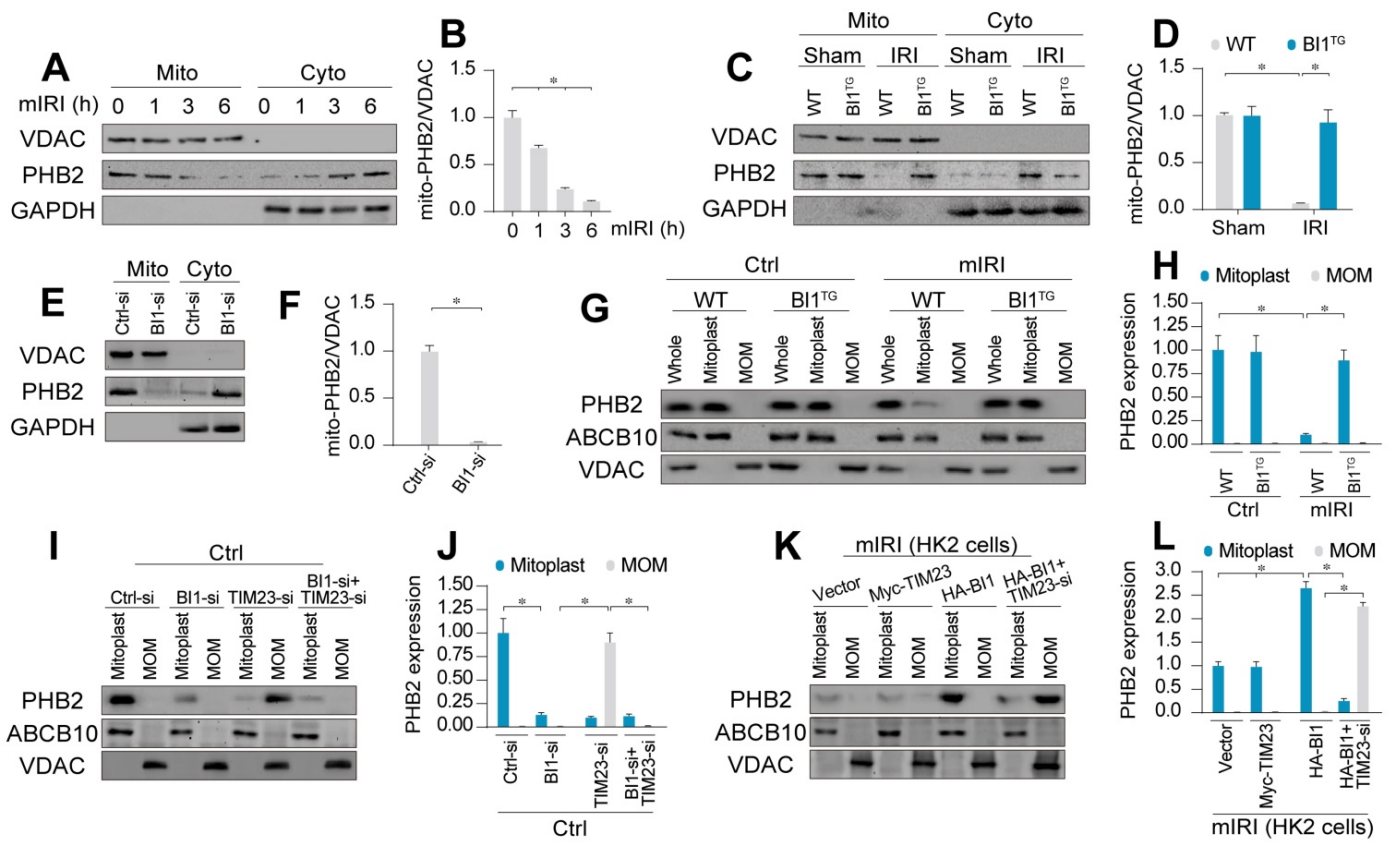
** BI1 promotes import of PHB2 into mitochondria. (A-B)** *In vitro*, after different times of IRI, proteins were isolated from tubule cells. Then, mitochondrial and cytosolic fractions were collected. PHB2 expression was determined using Western blots. VDAC was employed as the loading control for mitochondrial fraction whereas GAPDH was used as the marker of cytosolic fraction. **(C-D)** *In vivo*, proteins were isolated from reperfused kidney and mitochondrial and cytosolic fractions were collected. PHB2 expression was determined using Western blots. VDAC was utilized as the loading control for mitochondrial fraction whereas GAPDH was employed as the marker of cytosolic fraction. **(E-F)** siRNA against BI1 (BI1-si) and control siRNA (Ctrl-si) were transfected into primary tubule cells and then the expression of mitochondrial PHB2 (mito-PHB2) was determined. **(G-H)** In primary tubule cells from *BI1*^TG^ and WT mice, whole mitochondrial fraction (Whole) was firstly isolated and then mitochondrial outer-membrane (MOM) and mitoplast (inner-membrane plus matrix) fractions were collected. Western blotting was used to analyze the expression of PHB2 in whole, mitoplast and MOM fractions. ABCB10 was utilized as a loading control for mitoplast whereas VDAC was used as a MOM marker. **(I-J)** Under normal condition, BI1 siRNA (BI1-si), TIM23 siRNA (TIM23-si) and control siRNA (Ctrl-si) were transfected into primary tubule cells. Then, levels of PHB2 were determined. **(K-L)** Under mIRI condition, Myc-TIM23, HA-BI1 and vector were transfected into HK2 cells. Moreover, TIM23-si was employed to silence TIM23 in HK2 cells infected with HA-BI1 prior to determination of PHB2. Experiments were repeated for at least three times and data are shown as mean ± SEM (n = 6 mice or 3 independent cell isolations per group). *p<0.05.

Earlier evidence has depicted a possible cross-linking between BI1 and PHB2 13 - a newly identified mitochondrial receptor for mitochondrial integrity, bioenergetic metabolism, and quality control 17. Hence, we went on to examine if PHB2 was permissive to BI1-mediated mitochondrial benefit. qPCR assay demonstrated that PHB2 transcription remained unchanged following IRI in both WT and *BI1^TG^* mice (Figure S3A). Similarly, neither IRI nor *BI1* reconstitution affected the expression of total PHB2 (Figure S3B-C). Given that mitochondrial PHB2 compartmentalization is necessary to preserve mitochondrial integrity and function 23, mitochondrial proteins were isolated to evaluate PHB2 levels in subcellular compartments. Data presented in Figure 3A-B revealed that the levels of mitochondrial PHB2 (mito-PHB2) were downregulated whereas cytoplasmic PHB2 (cyto-PHB2) content was upregulated in response to various durations of mIRI challenge. To understand the role of mitochondria-localized PHB2, HK2 tubule cell lines were transfected with PHB2 mutant lacking the N-terminal mitochondrial targeting domain (PHB2ΔN) which restricts PHB2 from importing into mitochondria. Meanwhile, PHB2 mutant lacking C-terminal domain (PHB2ΔC) was used as the control group. After transfection with PHB2ΔN but not PHB2ΔC, cell viability was reduced (Figure S3D), a result that occurred with a rise in caspase-9 activity (Figure S3E), suggesting that mitochondria-localized PHB2 is necessary to preserve tubule cell survival and mitochondrial function.

Although IRI mediated PHB2 export from mitochondria into cytoplasm, *BI1* reconstitution effectively reversed the changes in mito- and cyto-PHB2 in the face of IRI (Figure 3C-D). The regulatory role of BI1 on PHB2 mitochondrial localization was further consolidated through a loss-of-function assay using BI1 siRNA (BI1-si). In normal cells, BI1 knockdown greatly reduced mito-PHB2 levels (Figure 3E-F), although PHB2 transcription remained constant (Figure S3F).

Structurally, PHB2 is a mitochondrial inner membrane (MIM) protein. To elucidate the scenario behind BI1-provoked PHB2 retention onto MIM, digitonin extraction technique was employed to separate mitoplast (MIM plus matrix fractions) and mitochondrial outer membrane (MOM). ABCB10 and VDAC were utilized as markers of mitoplast and MOM, respectively. Under normal condition, PHB2 was only detectable in mitoplast but not MOM fractions (Figure 3G-H). Following mIRI, levels of PHB2 in mitoplast were downregulated, an effect that was reversed in tubule cells isolated from *BI1^TG^* mice, denoting a role for BI1 in PHB2 sequestration onto MIM.

There are two steps for PHB2 import into MIM: first shuttle from cytoplasm onto the MOM and then transport across the MOM to MIM with the assistance of TIM23 44, a component of the mitochondrial protein import system. To elucidate how BI1 controls PHB2 translocation from cytoplasm into MIM, BI1 and TIM23 were silenced prior to re-examination of the localization of PHB2 in mitoplast and/or MOM. Under physiological condition, only mitoplast but not MOM PHB2 was detected (Figure 3I-J). Upon deletion of BI1, levels of mitoplast PHB2 were downregulated without PHB2 localization in MOM (Figure 3I-J). To our surprise, TIM23 knockdown greatly lowered the levels of PHB2 in mitoplast and promoted PHB2 accumulation in MOM. Interestingly, TIM23 deficiency-associated PHB2 accumulation in MOM was nullified by BI1 silencing (Figure 3I-J). This result suggested that BI1 is required for PHB2 localization onto MOM whereas PHB2 transport across MOM into MIM is mainly governed by TIM23. This finding was further confirmed in HK2 cells using co-transfection of HA-BI1 and Myc-TIM23. Upon exposure to mIRI, HA-BI1 rather than Myc-TIM23 upregulated the levels of PHB2 in mitoplast (Figure 3K-L). However, HA-BI1 could only promote PHB2 aggregation in MOM fraction in the absence of TIM23. In sum, BI1 facilitates PHB2 localization onto MOM and this process serves as an initial step for the import of PHB2 into mitochondria.

### BI1 interacts with PHB2 and augments PHB2 mitochondrial localization

**Figure 4:**
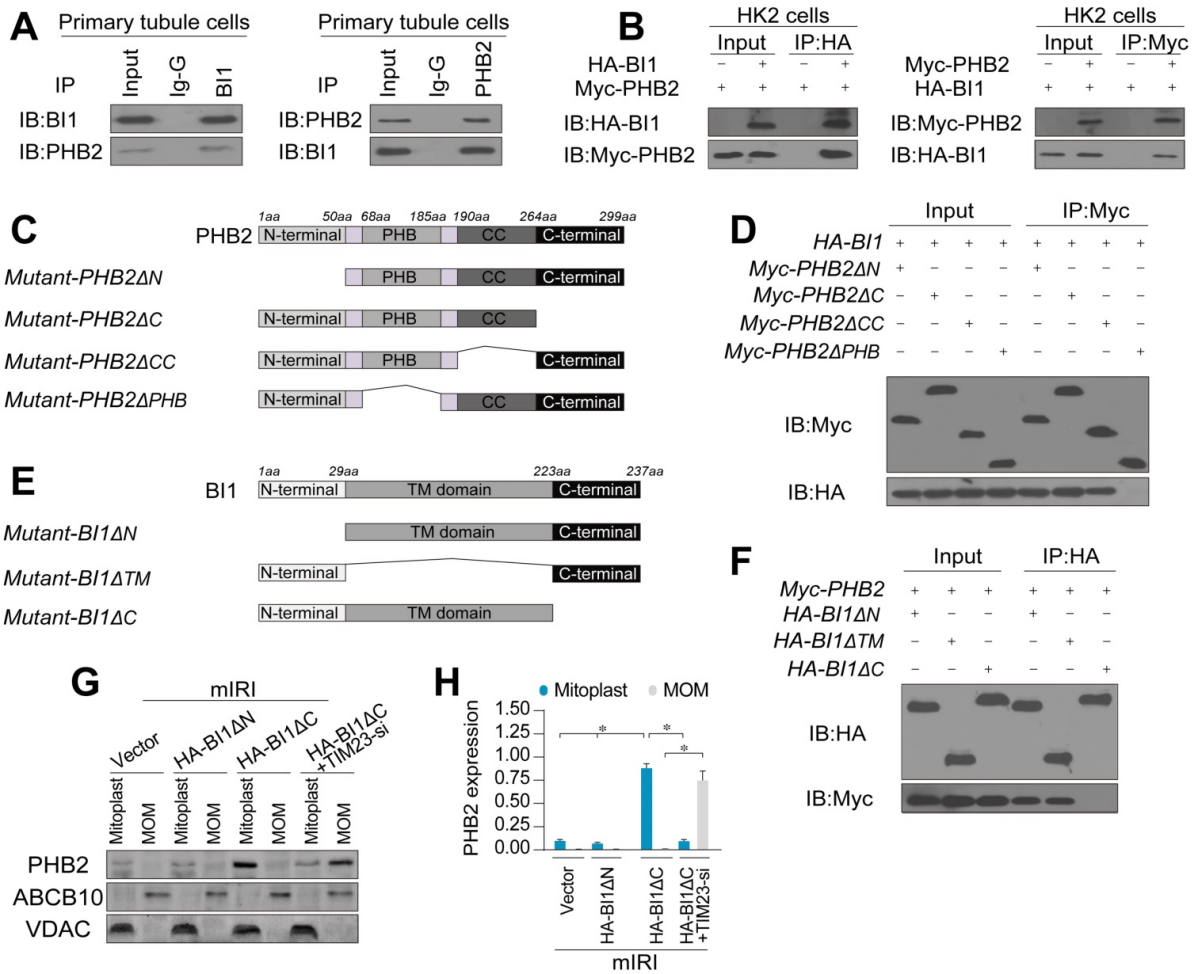
** BI1 interacts with PHB2 and promotes PHB2 localization into mitochondria under renal IRI. (A)** Cell lysates from primary tubule cells were immunoprecipitated with the anti-BI1 or anti-PHB2 antibody, followed by immunoblotting with the anti-PHB2 or anti-BI1 antibody. IgG was employed as a control for the endogenous interaction assay between BI1 and PHB2. **(B)** Immunoblotting analysis of lysates after immunoprecipitation from HK2 cells transfected with exogenous HA-BI1 and Myc-PHB2.** (C-D)** Mapping of regions of PHB2. Different PHB2 mutants were transfected into HK2 cells. Then, immunoprecipitation, and immunoblot of cell lysates from HK2 cells. **(E-F)** Different BI1 mutants as indicated were transfected into HK2 cells, and then immunoprecipitation analyses were carried out. **(G-H)** Under mIRI, different BI1 mutants were transfected into HK2 cells. Besides, TIM23-siRNA was utilized to silence TIM23 in HK2 cells transfected with Myc-BI1 mutants. Experiments were repeated for at least three times and data are shown as mean ± SEM (n =3 independent cell isolations per group). *p<0.05.

To further discern the mechanism through which BI1 promotes PHB2 translocation onto MOM, we examined the possibility whether BI1 interacts with PHB2. Co-IP assay demonstrated a constitutive interaction between endogenous BI1 and PHB2 in primary tubule cells (Figure 4A). In line with these findings, exogenous interaction between BI1 and PHB2 was also observed in HK2 tubule cell lines that were co-transfected with Myc-PHB2 and HA-BI1 (Figure 4B). To further explore the molecular basis of the interaction between BI1 and PHB2, we analyzed the regions of BI1 and PHB2 that are required for cross-linking. First, a series of PHB2 deletion mutants were transfected into HK2 cells (Figure 4C). Co-IP assay demonstrated that the mutant lacking the PHB domain fully nullified its ability to interact with BI1 (Figure 4D). On the other side of the coin, BI1 possesses several transmembrane domains with both N and C termini in the cytosol and thus three kinds of BI1 domain-deletion mutants were generated (Figure 4E). BI1 protein lacking the N-terminal domain (ΔN) or TM domain (ΔTM) could interact with PHB2 whereas BI1 lacking the C-terminal domain (ΔC) interrupted the binding of BI1 and PHB2 (Figure 4F). This evidence indicates that the PHB domain in PHB2 and the C-terminal region of BI1 are obligatory for the cross-linking between PHB2 and BI1.

To understand whether BI1/PHB2 interaction is required for PHB2 mitochondrial retention, BI1 mutants were transfected into HK2 cells in the presence of mIRI. Then, mitoplast and MOM fractions were isolated for western blots. As expected, compared to the mIRI-treated cells, HA-BI1ΔN rather than HA-BI1ΔC mutant stabilized the expression of PHB2 in mitoplast (Figure 4G-H), suggesting that interruption of BI1/PHB2 interaction led to a failure of PHB2 import into mitochondria. Moreover, in the absence of TIM23, HA-BI1ΔN only promoted PHB2 aggregation in MOM fraction (Figure 4G-H), reconfirming the notion of BI1/PHB2 interaction in promoting PHB2 docking onto MOM.

### PHB2 mitochondrial localization accounts for BI1-mediated mitochondrial protection

**Figure 5:**
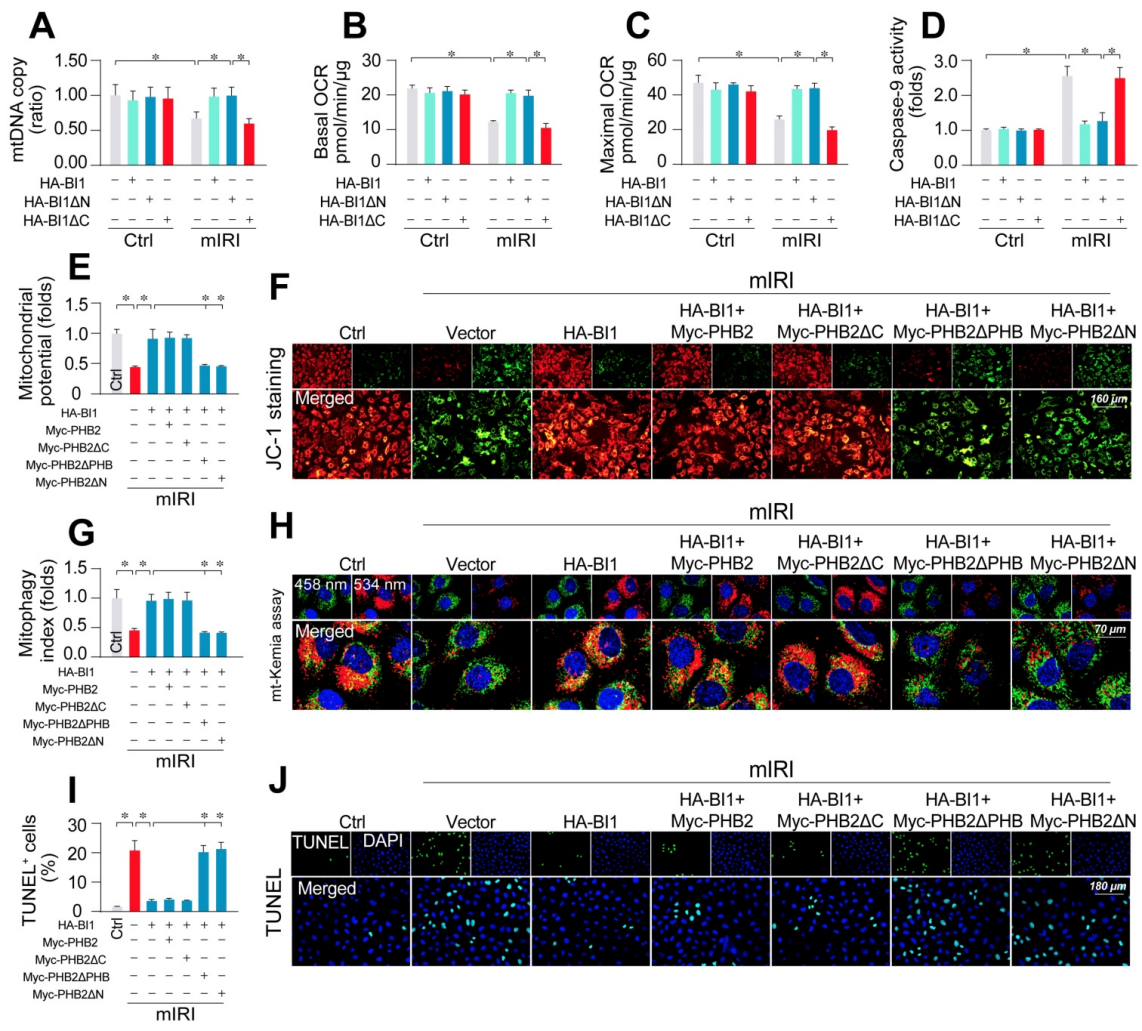
** PHB2 retention in mitochondria accounts for BI1-conferred renoprotection. (A)** Prior to mIRI, HK2 cells were transfected with HA-BI1 or its mutants (HA-BI1ΔC and HA-BI1ΔN). Mitochondrial copy number was determined using qPCR. **(B-C)** Mitochondrial OCR was determined using an XFe96 extracellular flux analyzer in HK2 transfected with HA-BI1 and/or its mutants. **(D)** ELISA for caspase-9 activity. **(E-F)** Myc-labelled PHB2 mutants (Myc-PHB2ΔPHB, Myc-PHB2ΔC, Myc-PHB2ΔN) were infected into HK2 cells. Besides, HA-BI1 or vector were constructed into HK2 cells prior to mIRI. Mitochondrial membrane potential was recorded using JC-1 staining. **(G-H)** mt-Kemia assay for acidic mitochondria observation. The ratio of 534/458 nm was used to quantify acidic mitochondria index. **(I-J)** TUNEL assay for cell death. Number of TUNEL apoptotic cells were calculated. Experiments were repeated for at least three times and data are shown as mean ± SEM (n = 3 independent cell isolations per group). *p<0.05.

To better explicate whether BI1-induced PHB2 mitochondrial retention is necessary to preserve mitochondrial function, HK2 cells were transfected with BI1 mutants. Upon mIRI exposure, mtDNA copy (Figure 5A), transcription (Figure S4A), ETC activity (Figure S4B-C), mitochondrial respiration (Figure 5B-C), and mitochondrial survival (Figure 5D) were preserved by HA-BI1 or HA-BI1ΔN but not HA-BI1ΔC. Besides, PHB2ΔPHB transfection also abrogated BI1-mediated mitochondrial genomic protection under mIRI (Figure S5A-D). In addition, BI1 overexpression sustained mitochondrial membrane potential (Figure 5E-F), preserved mitophagy (Figure 5G-H), and inhibited tubule cell death (Figure 5I-J) although such effect was absent with the transfection of Myc-PHB2ΔPHB. Given that the N-terminal mitochondrial targeting domain of PHB2 is required for PHB2 docking onto mitochondria 21, PHB2ΔN (which cannot be localized into mitochondria) was used as a positive control group and it also blunted BI1-mediated mitochondrial protection after exposure to mIRI. In sum, BI1 confers PHB2 retention in mitochondria leading to preservation of mitochondrial integrity and prevention of tubular apoptosis under IRI.

### Deletion of PHB2 abolishes BI1-offered renoprotection* in vivo*

**Figure 6:**
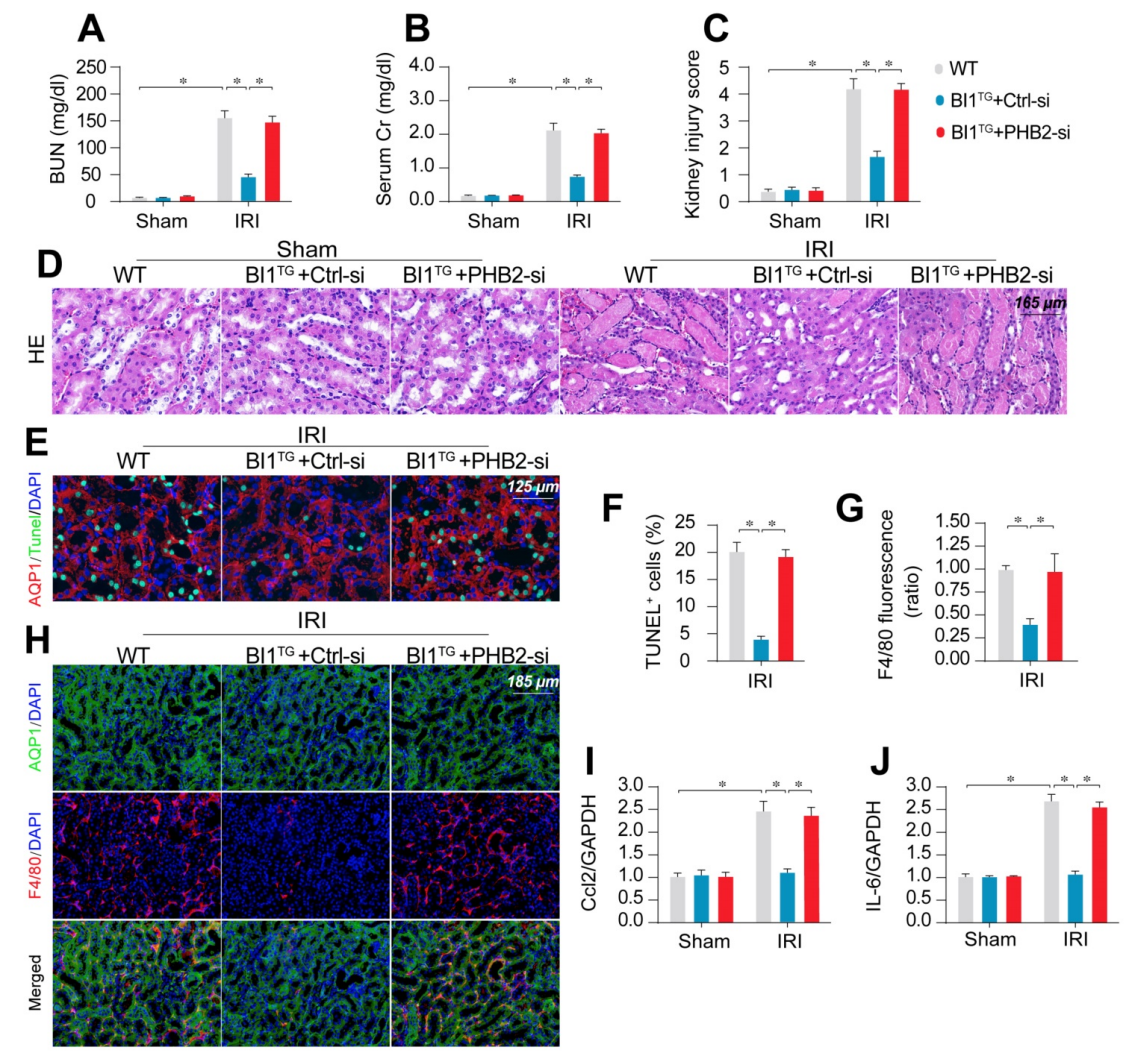
** PHB2 knockdown abolishes BI1-induced renoprotection*.* (A-B)** *BI1*^TG^ mice were subjected to intravenous injections of scramble control or PHB2-specific siRNA (Ctrl-si or PHB2-si) before IRI. Then, renal function was determined using levels of BUN and creatinine. **(C-D)** HE staining was used to observe the structural alterations of tubules following IRI or the whole kidney. Semiquantitative analysis of tubular injury (tubular atrophy or dilatation, loss of brush border, vacuolization, epithelial cell shedding, and denuded tubular basement membrane) scored as: 0, normal; 1, <10%; 2, 10%-25%; 3, 25%-50%; 4, 50%-75%; 5, 75%-100% of affected area from 20 random fields. **(E-F)** Tubule death was detected using TUNEL staining. AQP1 was employed to stain proximal tubules. **(G-H)** Immunofluorescence assay for F4/80 inflammatory cells. The immunosignal of F4/80 was used to evaluate kidney inflammation response. **(I-J)** RNA was isolated from reperfused kidneys and then transcriptional levels of Ccl2 and IL-6 were determined using qPCR. Experiments were repeated for at least three times and data are shown as mean ± SEM (n = 6 mice or 3 independent cell isolations per group). *p<0.05.

To transpose our *in vitro* findings into the *in vivo* animal model, WT and *BI1^TG^* mice were subjected to daily intravenous injection of control or PHB2 siRNA (Ctrl-si or PHB2-si) three days prior to IRI challenge. In line with findings noted previously, renal failure (Figure 6A-B), tubule damage (Figure 6C-D), cell death (Figure 6E-F), and kidney inflammation (Figure 6G-J) caused by IRI were markedly mitigated in *BI1^TG^* mice. However, these beneficial changes of *BI1^TG^* mice were no longer prevailed with PHB2-si injection. *In vitro* data suggested that PHB2 silencing abolished the anti-apoptotic (Figure S6A-B) and anti-inflammatory (Figure S6C-D) properties of BI1 in tubule cells under mIRI.

## Discussion

**Figure 7:**
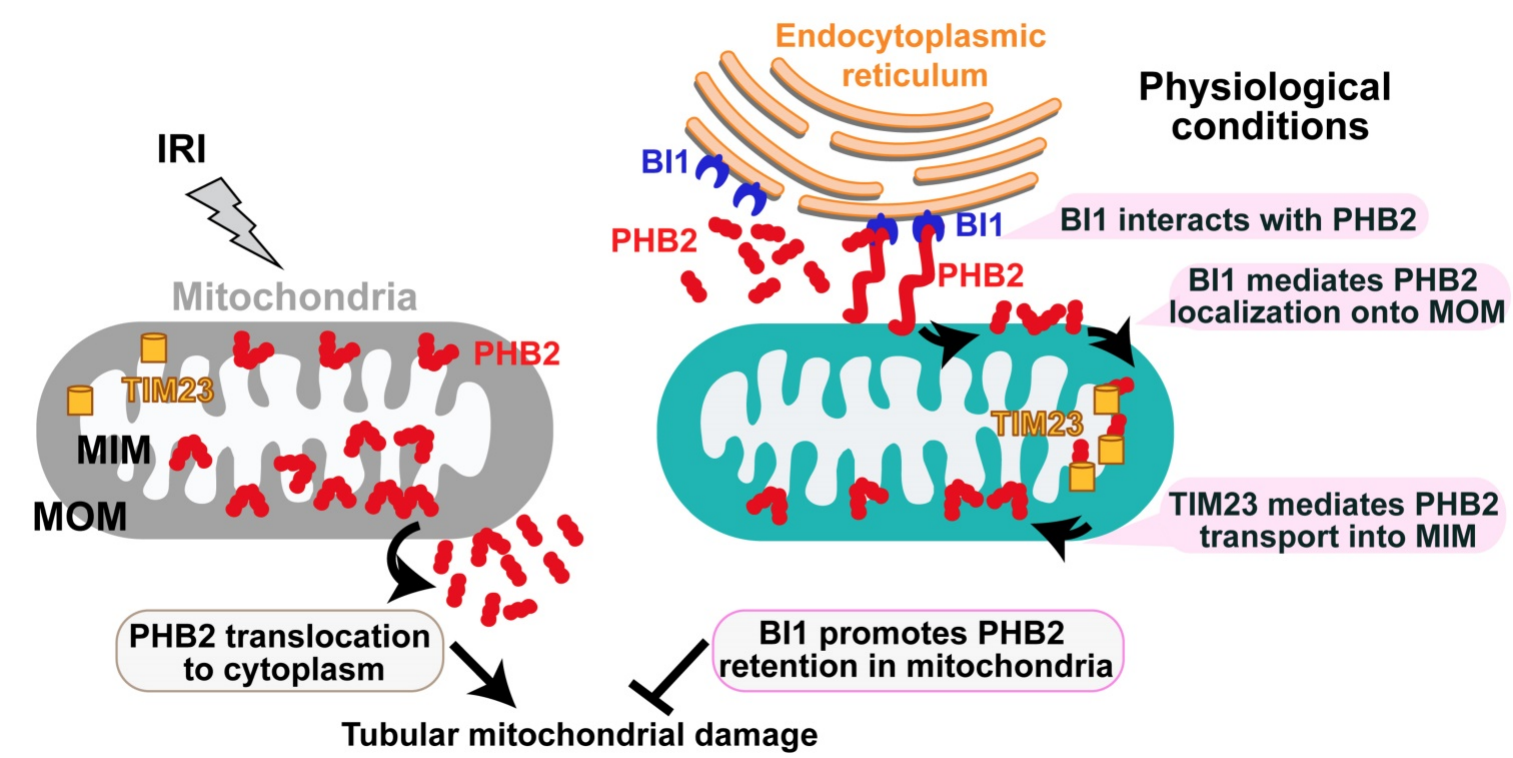
Schematic diagram depicting proposed BI1-PHB2 signaling modality in AKI. In physiological settings (as shown in the right panel), BI1 interacts with and therefore promotes PHB2 retention into mitochondria with the assistance of the mitochondrial transport protein TIM23, preserving mitochondrial homeostasis and tubular viability. Pathological stress such as IRI (as shown on the left panel) suffers from loss of BI1, leading to poor MOM localization and translocation of PHB2 into MIM. As a result, PHB2 is lost into cytoplasm (loss of mitochondrial retention) to trigger mitochondrial damage.

Our data presented here have provided convincing molecular mechanisms for tubular mitochondrial injury in the setting of AKI. We have shown for the first time that: 1) BI1, as an indispensable inhibitor of Bax-related mitochondrial apoptosis, is downregulated in both urine and plasma throughout the course of AKI; 2) reconstitution of BI1 protects renal function against AKI through attenuating mitochondrial damage; 3) mechanically, BI1 interacts with PHB2 and promotes the import of cytoplasmic PHB2 into mitochondria with the assistance of TIM23; 4) mitochondria-localized PHB2 sustains mitochondrial morphology/function and represses mitochondrial apoptosis; and 5) absence of PHB2 abolishes BI1 reconstitution-mediated renoprotection both *in vivo* and *in vitro* (Figure 7). These results offer evidence for the first time that BI1, through sustaining mitochondrial function and tubular survival by way of PHB2 retention into mitochondria, may represent a critical mitochondrial apoptotic regulatory target in the management of AKI.

In our study, the levels of BI1 in urine, urinary sediments and plasma from AKI patients were reduced through an undefined mechanism. Interestingly, BI1 reconstitution confers resistance for tubule cells to AKI-induced mitochondrial damage. Consistent with previous studies 45-48, the protective mechanisms exerted by BI1 in AKI involves elimination of cellular ROS, interruption of mitochondrial fission, and inhibition of mitochondrial apoptosis. Besides, based on our results, BI1 also plays unforeseen roles in sustaining mtDNA copy/transcription, mitochondrial respiration and mitophagy. Mitochondrial genomic stability is associated with the functionality of ETCx whereas mitophagy degradation system is employed to timely remove the unhealthy mitochondria mass. These mitochondrial protective features of BI1, in conjunction with the fact that BI1 could be detected in urine sediments and plasma in human, might provide a potential target for clinical monitoring and therapeutic evaluation of AKI.

Perhaps the most intriguing finding from our study is that BI1 reconstitution-induced mitochondrial and renal preservation are accomplished through upregulation of mitochondria-localized PHB2. PHB2 dissociation from mitochondria and translocation into cytoplasm/nucleus have been acknowledged as a trigger for mitochondrial apoptosis 49. In fact, maintenance of mitochondria-localized PHB2 is a dynamic process, which is determined by a ratio between mitochondrial PHB2 import and export. At baseline, PHB2 is primarily localized on mitochondria and partially resides in cytoplasm. Following exposure to AKI, the level of mito-PHB2 is reduced whereas cyto-PHB2 expression is augmented. Thereby, PHB2 mitochondrial import may be considered a physiological process whereas the PHB2 “escape” from mitochondria could be considered as an injury response to AKI. In the setting of AKI, BI1 reconstitution is capable of reversing levels of mito-PHB2, suggesting that BI1 may help to preserve physiological PHB2 mitochondrial translocation. Although PHB2 deficiency has been suggested to prompt mitochondrial damage and kidney dysfunction 24, 25, 50, our study provides a novel insight for the mitochondria-localized PHB2, rather than total PHB2, in mitochondrial quality control. Of note, BI1 was originally reported to be a safeguard for MOM homeostasis through blocking Bax-mediated MOM permeabilization. Our current findings have unveiled its new role in MIM management via sustaining PHB2 mitochondrial retention, part from the classical modality of MOM regulation.

Mechanically, we found that BI1 directly interacts with the cytoplasmic PHB2 and facilitates PHB2 sequestration onto MOM. Then, with the assistance of TIM23, PHB2 gets translated from MOM and finally settles onto MIM whereas it exerts pronounced mitochondrial protective effects and participates in BI1-mediated renoprotection in the face of AKI. Structural analysis illustrated that the C-terminal domain of BI1 is required for the BI1/PHB2 (through PHB domain) interaction. Although C-terminal domain of BI1 was originally considered a key region to inhibit Bax-related mitochondrial apoptosis^51^, BI1 may also utilizes its C-terminal region to interact with NADPH-dependent cytochrome P450 reductase^52^, ATG6^53^, 1,4,5-trisphosphate (IP3) receptor (IP3R)^54^, and presenilin 1^55^. In addition to BI1, our findings verified that the PHB domain on PHB2 protein structure is also involved in the BI1/PHB2 cross-linking. The PHB domain in PHB2, also known as the SPFH (stomatin, prohibitin, flotillin, and HflC/K) domain, is essential for protein interaction with estrogen receptor, tyrosine phosphatase-1^56^ and the autophagy initiator LC3^57^. The likely interaction between BI1 and PHB2 noted in our current study is somewhat different from the inter-organelle communication between ER and mitochondria 58. Several mitochondrial pathophysiological processes are temporally and spatially linked to ER, such as fission 59, calcium recycling 60, ROS production^61^, and apoptosis^62^. However, findings revealed in our current study did not favor direct interaction between ER and mitochondria for BI1-PHB2 interaction. Rather, our finding describes a different and novel role offered by the ER-localized BI1 in stabilizing mitochondria-localized PHB2 through direct interaction with cytoplasmic PHB2 prior to its import into mitochondria. These observations should help to uncover a striking pattern of mutual interaction between the ER anti-apoptotic signaling and mitochondrial quality control.

Several limitations exist in our present study. First and perhaps the foremost, it remains essentially unknown for the cellular and molecular basis behind BI1 downregulation in AKI or IRI stress. Post-transcriptional modification and/or protein degradation machinery may play a role in the loss of BI1 expression. Second, the PHB2 disassociation from mitochondria seems to be regulated via an undefined mechanism. Collectively, BI1 protects tubular mitochondria against renal IRI through a direct interaction with PHB2. The BI1-PHB2 modality for mitochondrial protection reported here in our study should shed some promises towards novel therapeutic targeting in the clinical management of AKI.

## Supplementary Material

Supplementary figures and tables.
