# Supplementary material for: Bax inhibitor 1 preserves mitochondrial homeostasis in acute kidney injury through promoting mitochondrial retention of PHB2
Source: Theranostics. 2020 Jan 1;10(1):384–97. doi: 10.7150/thno.40098 (PMC6929616; doi:10.7150/thno.40098)
Supplement: Supplementary file 1 — Supplementary figures and tables. [file thnov10p0384s1.pdf]

Supplemental figures and tables for

Bax inhibitor 1 preserves mitochondrial homeostasis in acute kidney injury through promoting mitochondrial retention of PHB2

Jin Wang[1*], Pingjun Zhu[1*], Ruibing Li[1], Jun Ren[2,3#], Yingmei Zhang[2,3#], Hao Zhou[1,2#]

[1]Chinese PLA General Hospital, Medical School of Chinese PLA, Beijing, 100853, China;

[2]Center for Cardiovascular Research and Alternative Medicine, University of Wyoming College of Health Sciences, Laramie, WY 82071 USA

[3]Department of Cardiology and Shanghai Institute of Cardiovascular Diseases, Zhongshan Hospital, Fudan University, Shanghai, China 200032

Running title: BI1 attenuates AKI-related mitochondrial damage

[*]The first two author contributed equally to this article.

[#]Correspondence to:

Dr. Jun Ren (E-mail: jren@uwyo.edu), Dr. Yingmei Zhang (E-mail: zhangym197951@126.com) or Dr. Hao Zhou (E-mail: zhouhao301@outlook.com)

Center for Cardiovascular Research and Alternative Medicine, University of Wyoming College of Health Sciences, Laramie, WY 82071, USA.

Tel: (307) 766-6131; Fax: (307) 766-2953

Supplemental Figure 1: BI1 modulates IRI-related renal damage. A-B. HK2 cells were transfected with siRNA against BI1 (BI1-si) and Control siRNA (Ctrl-si). Then, western blot was used to detect the expression of BI1. **C.** Primary tubule cells were isolated from $BI1^{TG}$ and WT mice. The mimicked IRI (mIRI) model was employed through rotenone (10 mM) in glucose-free DMEM for 3-h followed by 3-h full culture medium incubation. Then, cell viability was determined using MTT assay. **D-E.** RNA was isolated from mIRI-treated cells and transcriptions of Ccl2 and IL-6 were used to evaluate tubule damage. Experiments were repeated for at least three times and data are shown as mean ± SEM (n = 6 mice or 3 independent cell isolations per group). *p<0.05.

Supplemental Figure 1

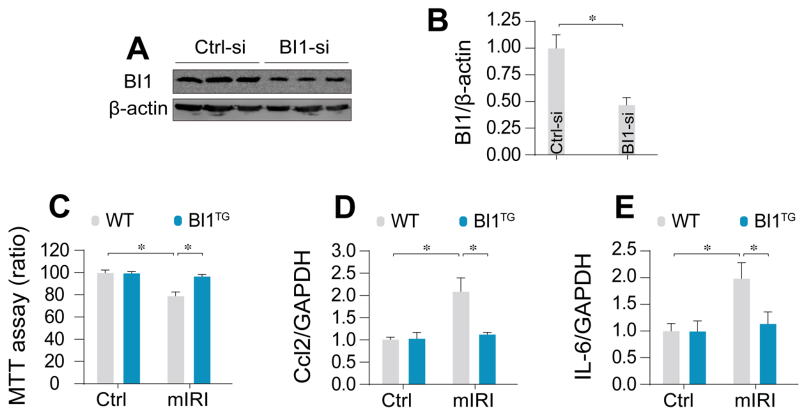

Supplemental Figure 2: Mitochondrial homeostasis is sustained in response to BI1 overexpression under kidney IRI. A-B. The transcription of mitochondrial DNA was determined via qPCR. **C-D.** ELISA was used to analyze the activity of mitochondrial respiratory complex I and II. **E-F.** *In vitro*, RNA was isolated from cells and levels of PGC1α and Sirt3 were measured via qPCR. **G.** Mitochondrial morphology was observed using electron microscope. Yellow arrows demonstrated elongated mitochondrial tubules and red arrows indicated swollen mitochondria with fractured cristae. **H.** Proteins were isolated from tubule cells after *in vitro* mIRI and level of mitochondrial apoptosis was determined using Western blotting. Experiments were repeated for at least three times and data are shown as mean ± SEM (n = 6 mice or 3 independent cell isolations per group. *p<0.05.

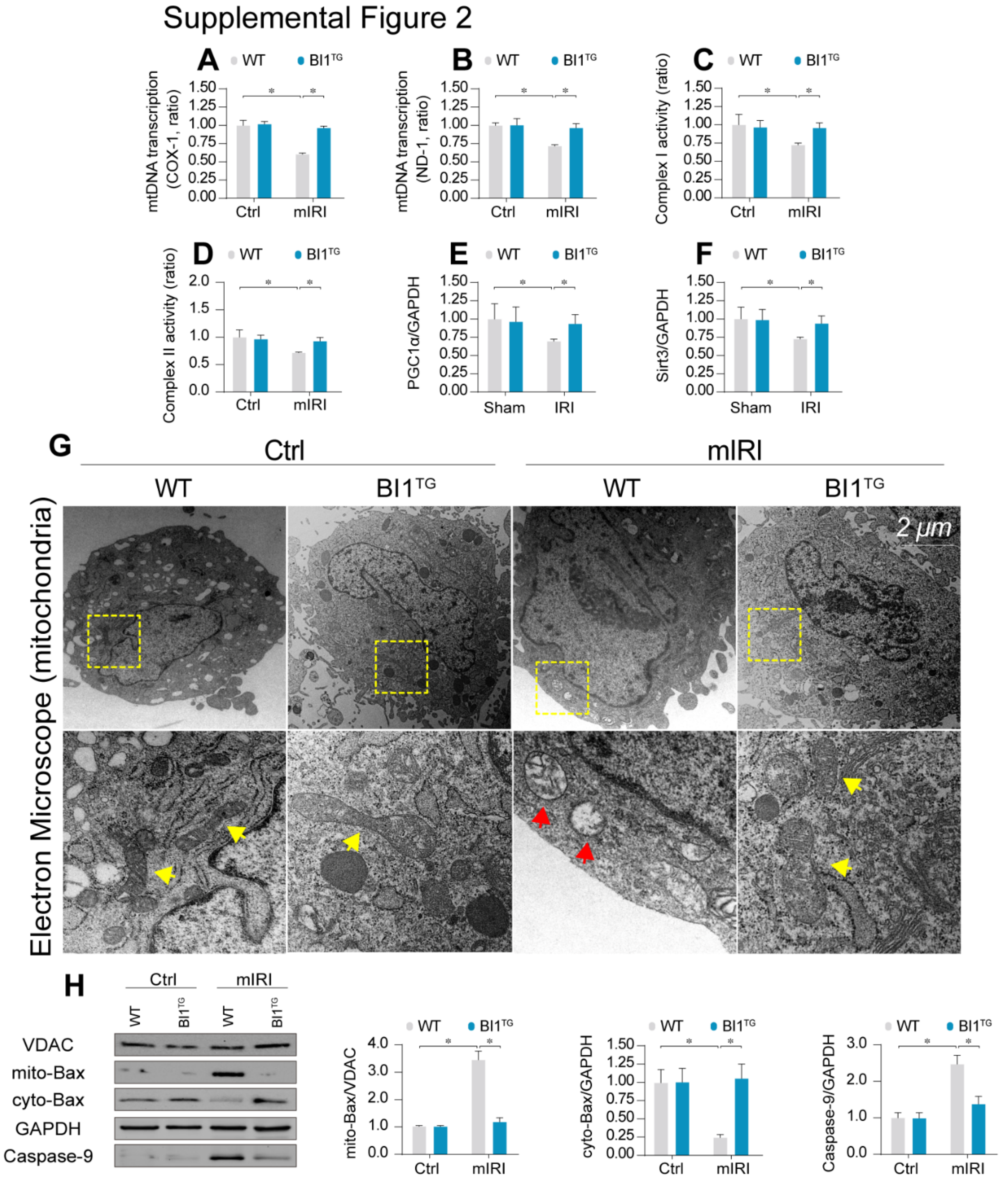

Supplemental Figure 3: BI1 induces PHB2 localization into mitochondria. A. RNA was isolated from IRI-treated kidneys and transcription of PHB2 was determined using qPCR. **B-C.** Western blotting for PHB2 in *BI1*^{TG} and WT mice under IRI. **D.** Myc-labelled PHB2 mutants lacking N domain (Myc-PHB2ΔN) or C-terminal domain (Myc-PHB2ΔC) were infected into HK2 cells. Then, cell viability was determined via MTT assay. **E.** Caspase-9 activity was measured to evaluate the influence of PHB2 mutants on mitochondrial function. **F.** *In vitro*, siRNA against BI1 (BI1-si) and control siRNA (Ctrl-si) were transfected into primary tubule cells and then the transcription of PHB2 was determined via qPCR. Experiments were repeated for at least three times and data are shown as mean ± SEM (n = 6 mice or 3 independent cell isolations per group). *p<0.05.

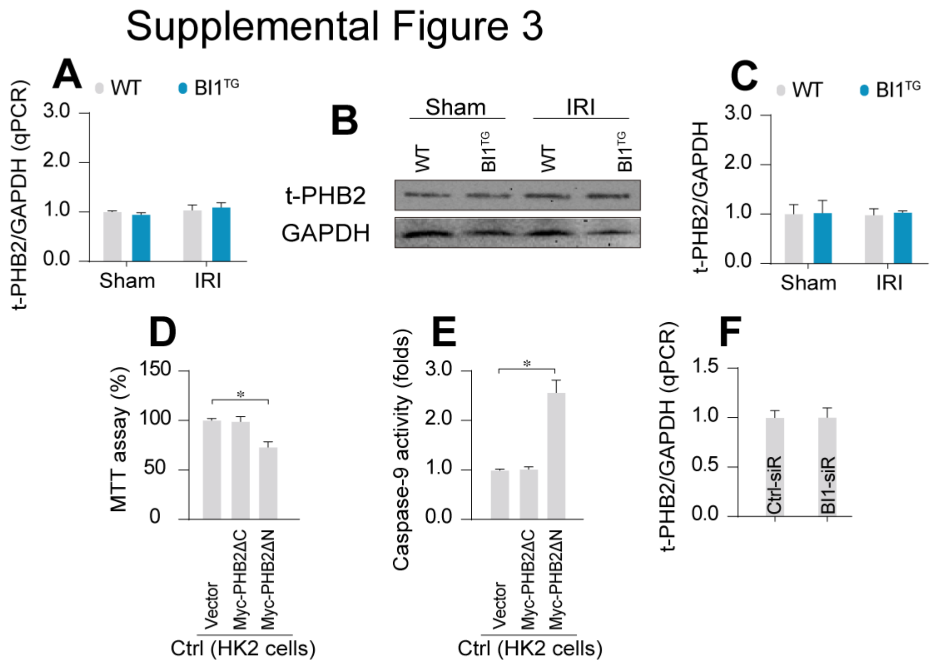

Supplemental Figure 4: PHB2 is required for BI1-mediated mitochondrial protection. A. Prior to mIRI challenge, HK2 cells were transfected with HA-BI1 and its mutants (HA-BI1ΔC and HA-BI1ΔN). Subsequently, mitochondrial DNA transcription was detected using qPCR. **B-C.** ELISA was used to analyze the activity of mitochondrial respiratory complex I and II. Experiments were repeated for at least three times and data are shown as mean ± SEM (n = 3 independent cell isolations per group). *p<0.05.

Supplemental Figure 4

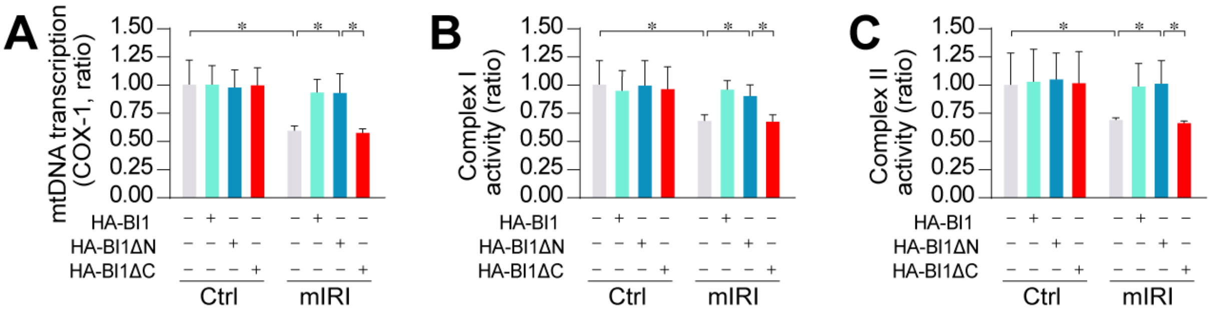

Supplemental Figure 5 Mitochondria-localized PHB2 accounts for BI1-mediated mitochondrial protection. A-B. Myc-labelled PHB2 mutants (Myc-PHB2ΔPHB, Myc-PHB2ΔC, Myc-PHB2ΔN) were infected into HK2 cells. HA-BI1 or vector were constructed into HK2 cells prior to mIRI challenge. Subsequently, mitochondrial DNA copy and transcription were detected using qPCR. **C-D.** ELISA was used to analyze the activity of mitochondrial respiratory complex I and II. Experiments were repeated for at least three times and data are shown as mean ± SEM (n = 3 independent cell isolations per group). *p<0.05.

Supplemental Figure 5

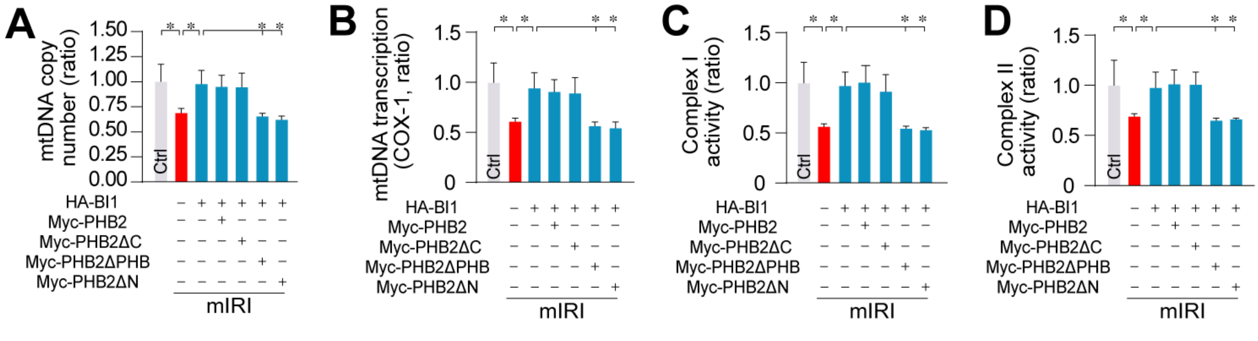

Supplemental Figure 6: PHB2 knockdown abolishes BI1-induced renoprotection. A. Primary tubule cells, isolated from WT and *BI1*^{TG} mice, were transfected with BI-si and Ctrl-si. Cell viability was determined using MTT assay. **B.** LDH release assay was used to measure cell death. **C-D.** RNA was isolated and transcriptions of Ccl2 and IL-6 were determined using qPCR. Experiments were repeated for at least three times and data are shown as mean ± SEM (n = 3 independent cell isolations per group). *p<0.05.

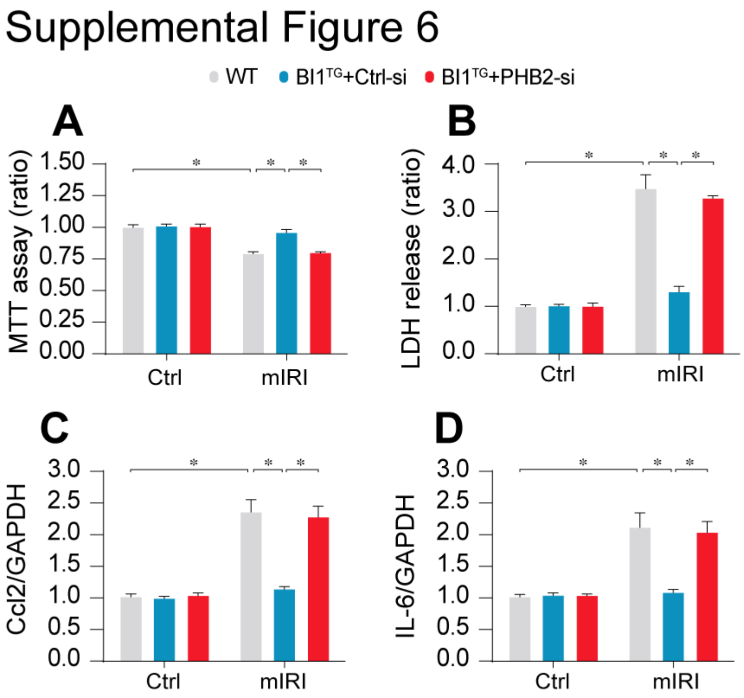

Supplemental Table 1 Patient demographics for AKI (-) and AKI (+) patients.

Patients characteristics	AKI (-) patients (n=27)	AKI (+) patients (n=28)
Age (range)	70 (43-87)	73 (45-86)
Sex	Male (n=18)	Male (n=21)
Comorbidities		
Hypertension (n)	7	9
Cardiovascular accident (n)	1	2
Diabetes (n)	3	2
Clinical data		
Acute tubular necrosis (n)	0	5
Sepsis (n)	3	6
Shock (n)	1	2
Acute coronary syndrome (n)	1	1
Hemorrhagic stroke	0	1
Paraquat intoxication	0	0

AKIN stage (n)		
I	0	14
II	0	9
III	0	5
APACHE II score (Average)	12-24 (18.7)	15-36 (22.1)
Baseline creatinine (mg/dl; average)	0.8-2.3 (1.2)	0.8-3.8 (1.8)
Peak creatinine (mg/dl; average)	0.8-2.3 (1.2)	1.9-4.1 (2.3)

AKIN, Acute Kidney Injury Network; APACHE, Acute Physiology, Age, Chronic Health Evaluation.

Supplemental Table 2: Antibody information in Western blot

Name	Catalogue number	Dilution factor
IL6	Abcam, #ab7737	1:1000
Sirt3	Abcam, #ab86671	1:1000
VDAC	Abcam, #ab15895	1:1000
GAPDH	Abcam, #ab181602	1:1000
ABCB10	Abcam, #ab74815	1:1000
BI1	Abcam, #ab18852	1:1000
Bcl2	Cell Signaling Technology, #3498	1:1000
PHB2	Cell Signaling Technology, #14085	1:1000
Ccl2	Cell Signaling Technology, #2029	1:1000
Bax	Cell Signaling Technology, #2772	1:1000
Caspase9	Cell Signaling Technology, #9504	1:1000

Supplemental Table 3: Primers for qPCR

Gene	Forward Prime	Reverse Prime
Ccl2	5′-GTTGGCTCAGCCAGATGCA-3′	5′-AGCCTACTCATTGGGATCATCTTG-3′
IL6	5′-TGGCTAAGGACCAAGACCATCCAA -3′	5-AACGCACTAGGTTTGCCGAGTAGA-3′
PHB2	5′-CCATTGTTAATGAGGTGCTCAA -3′	5′CTTCGGATCAACAGGGACA-3′
PGC1a	5′-TCACACCCCAAGCCCTTTT-3′	5′-GTGGGCTTCAACCAGCTTTG-3′
Sirt3	5′-GGTGCCTAGTGAGAGTGAGTCCCC-3′	5′-TCGGGGCTGAAGAGGGAGAAGTC-3′
GAPDH	5′-ACGGCAAATTCAACGGCACAGTCA-3′	5′-TGGGGGCATCGGCAGAAGG-3′
Kim1	5′-ACATATCGTGGAATCACAACGAC-3′	5′-ACTGCTCTTCTGATAGGTGACA-3′
ND-1	5′-ATGGTCAGTCTGTCATGGTGGAAC-3′	5′-GCATAGCACAAGCAGCGACAAC-3′
COX-1	5′-GAAGAGACAGTGTTTCATGTGGTGT-3′	5′-TCCTGGGCCTTTCAGGAATA-3′
Complex-IV	5′-CAGGATTCTTCTGAGCGTTCTATCA-3′	5′-AATTCCTGTTGGAGGTCAGCA-3′
